# Rethinking Gleason pattern quantification in predicting metastasis: results of 20 years of follow‐up in the Rotterdam section of the European Randomized Study of Screening for Prostate Cancer

**DOI:** 10.1111/his.70052

**Published:** 2025-11-26

**Authors:** Lisa J Kroon, Sebastiaan Remmers, Ivo I de Vos, Charlotte F Kweldam, L Lucia Rijstenberg, Roderick C N van den Bergh, Monique J Roobol, Geert J L H van Leenders

**Affiliations:** ^1^ Department of Pathology Erasmus MC Cancer Institute, University Medical Centre Rotterdam The Netherlands; ^2^ Department of Urology Erasmus MC Cancer Institute, University Medical Centre Rotterdam The Netherlands; ^3^ Department of Pathology Maasstad Hospital Rotterdam The Netherlands

**Keywords:** Gleason score, metastasis, needle biopsy, prostate cancer, quantitative Gleason grade

## Abstract

**Introduction:**

The Gleason grading system for prostate cancer (PCa) is based on the proportions of Gleason patterns (GP) 3–5. While pure GP3 has minimal metastatic potential, it is unclear whether GP3 quantity in the presence of GP4 and GP5 affects oncological outcomes.

**Objective:**

To assess the predictive value of PCa biopsy GP lengths on long‐term metastasis‐free survival (MFS).

**Methods:**

Prostate biopsies of 1,881 men with screen‐detected PCa who participated in the Dutch part of the European Randomized Study of Screening for Prostate Cancer (ERSPC) between 1993 and 2007 were revised for GP 3–5 length. Multivariable Cox regression analyses were used to evaluate the relationship between GP lengths and MFS truncated at 20 years, adjusting for clinical‐tumour stage (cT), prostate‐specific antigen (PSA), percentage positive biopsies and the presence of invasive cribriform/intraductal carcinoma (CR/IDC).

**Results and limitations:**

On multivariable analysis, ≥cT2, PSA, percentage positive cores and absolute length of GP4 and GP5 were all significantly associated with MFS. The discriminative ability was improved by adding CR/IDC to the model. Total GP3 length was neither associated with MFS in the model with (hazard ratio [HR] 0.99, 95% confidence interval [CI] 0.97–1.00, *P* = 0.3) nor without CR/IDC (HR 0.98, 95% CI 0.96–1.01, *P* = 0.2). A limitation is the lack of targeted biopsies.

**Conclusion and clinical implications:**

GP3 length does not have an impact on the prediction of MFS in biopsies, once GP4/GP5 lengths are known. Although GP3 percentage is essential in Gleason grading, MFS is related to absolute GP4 and GP5 quantity rather than their proportion to GP3.

## Graphical abstract

Example of prostate biopsy absolute lengths and proportions, and their impact on ISUP Grade Group assessment. While the absolute length of Gleason pattern 4 in all three pictured biopsies is the same (3 mm), the absolute length of Gleason pattern 3 differs, which leads to counter‐intuitive proportion‐based Grade Groups. Metastasis‐free survival is related to absolute Gleason patterns 4 and 5 quantity rather than their proportion to Gleason pattern 3.

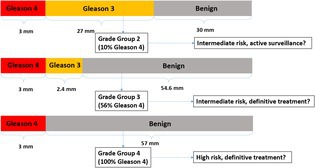

**Abbreviations**
CR/IDC: cribriform and/or intraductal carcinoma
CR/IDC: cribriform/intraductal carcinoma
cT: clinical‐tumour
ERSPC: European Randomized Study of Screening for Prostate Cancer
ERSPC: European Randomized Study of Screening for Prostate Cancer
GG: Grade Groups
GP: Gleason patterns
IQR: interquartile range
ISUP: International Society of Urological Pathology
MFS: metastasis‐free survival
MFS: metastasis‐free survival
MRI: magnetic resonance imaging
OCC: optimism corrected *c*‐index
PCa: prostate cancer
PSA: prostate‐specific antigen
RP: radical prostatectomy
TRUS: transrectal ultrasonography

## Introduction

The Gleason grading system is the cornerstone of pathological risk stratification, prediction of oncological outcomes and treatment decisions for prostate cancer (PCa) patients. Pathological grading of prostate biopsy and radical prostatectomy (RP) specimens is based on the assessment of three architectural categories with progressive glandular structural abnormality, known as Gleason patterns (GP). While GP1 and GP2 are not used in contemporary practice, relative proportions of GP3, GP4 and GP5 determine the Gleason score (GS). To support communication with patients, International Society of Urological Pathology (ISUP) Grade Groups (GG) categorize the GS into five comprehensive risk groups which should be reported in conjunction with the GS.^1^

**Figure 1:**
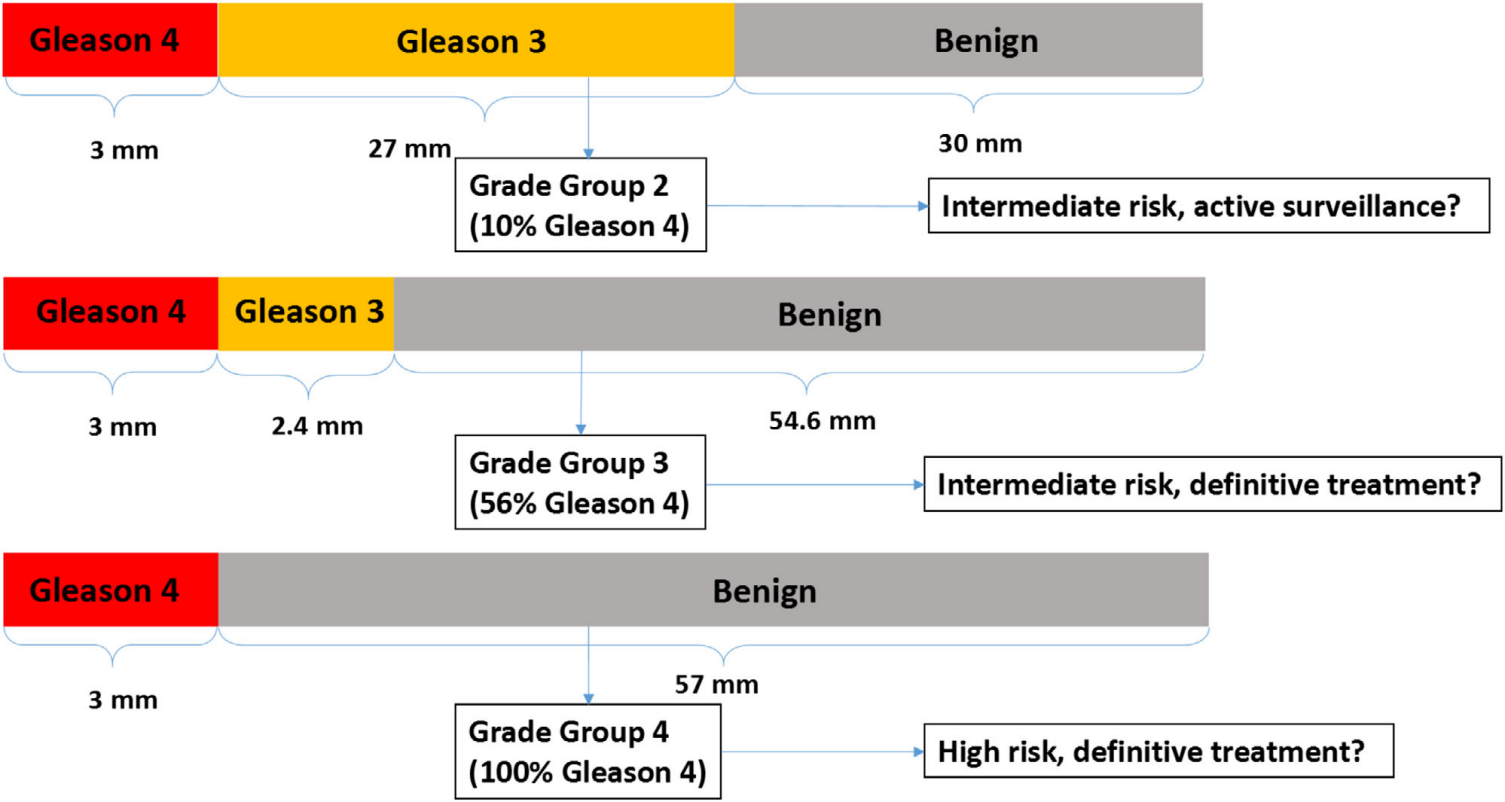
Example of prostate biopsy absolute lengths and proportions, and their impact on ISUP Grade Group assessment. While the absolute length of Gleason pattern 4 in all three pictured biopsies is 3 mm, the absolute length of Gleason pattern 3 differs, which leads to counterintuitive proportion‐based Grade Groups.

Last decades it has become clear that men with GG1 PCa at RP have an extremely low risk of developing metastasis or dying of disease.^2^, ^3^, ^4^ This has raised the question of whether GG1 should even be labelled as PCa at all.^5^, ^6^ While GP3 in GG1 disease only has very low, if any, capacity to metastasize, it is unclear what GP3's biological and clinical relevance is in higher GGs, where GP3 is present adjacent to GP4 and/or GP5. The decreasing percentage of GP3 in GS7 patients has strong predictive value for biochemical recurrence and metastasis‐free survival (MFS) in biopsy and RP specimens and underlies the separation of GS7 into 3 + 4 (GG2) with ≥50% GP3 and 4 + 3 (GG3) with <50% GP3.^7^, ^8^ However, the decreasing percentage of GP3 and simultaneous increase of GP4 in GS7 patients correlates with higher GP4 absolute volume.^9^, ^10^ Therefore, the actual prognostic value of GP3 quantity remains to be determined. Recently, Vickers *et al*. found that in GG2 patients, incorporating total GP3 length in biopsies into a multivariable model that included the volume of GP4, did not predict adverse surgical pathology.^9^ Additionally, current grading which depends on relative GP3, GP4 and GP5 proportions may lead to counterintuitive risk assessment. For instance, a 4‐mm tumour at biopsy with 60% (2.4 mm) GP4 and 40% (1.6 mm) GP3 is scored as GS 4 + 3 = 7 (GG3). On the other hand, a 12‐mm tumour with 60% (7.2 mm) GP3 and 40% (4.8 mm) GP4 is graded GS 3 + 4 = 7 (GG2). Although the GP4 volume in the latter case is twice as high as in the first example, its biopsy GG is lower. This contradictory finding can even be extended to low‐volume biopsy GG4 tumours with 100% GP4 and might have a major impact on clinical management (Figure 1).^11^

There is growing interest in the quantification of GP4 absolute length instead of percentage on biopsy specimens.^10^, ^11^, ^12^, ^13^, ^14^, ^15^, ^16^ However, many studies on quantitative grading lack long‐term outcomes and include selected cohorts excluding patients with GG4 or GG5. In addition, they do not evaluate invasive cribriform and/or intraductal carcinoma (CR/IDC), which are histopathological patterns associated with worse prognostic outcomes, especially metastasis.^17^

The objectives of this biopsy‐based study with long‐term follow‐up are (a) to analyse the correlation between GP4 absolute length and percentage, (b) to assess the predictive value of GP3 length on long‐term MFS, (c) to evaluate the discriminative ability of various GP quantification methods and (d) to assess the prognostic value of those methods if CR/IDC is considered.

## Methods

### Patient Selection

This retrospective study included men of the Dutch part of the European Randomized Study of Screening for Prostate Cancer (ERSPC) who had been diagnosed with PCa in one of the first three screening rounds between 1993 and 2007 in Erasmus Medical Centre, Rotterdam, The Netherlands. In the ERSPC, patients with elevated PSA levels (≥3.0 ng/mL) underwent systematic transrectal ultrasonography (TRUS)‐guided lateralized sextant biopsies. One or two additional lesion‐directed biopsies were taken if a hypoechoic lesion was present. The trial protocol has been described previously.^18^ The treatment of PCa, including salvage treatments, was performed according to Dutch policies and guidelines at the time of diagnosis. After initial treatment, patients were semi‐annually monitored by chart review to assess metastatic progression and secondary treatments. Men with metastases within 6 months from diagnosis were excluded from analysis because we aimed for long‐term prediction.

### Pathological Revision

All biopsies were revised by one genito‐urinary pathologist (GvL) who was blinded to patient information and outcome.^19^, ^20^ For each core, the GS, ISUP GG, GP3‐5 tumour percentage, CR/IDC, total tumour length and total biopsy length were assessed, according to the 2014 ISUP recommendations.^21^ IDC was defined according to the criteria of Guo and Epstein.^22^ Invasive cribriform carcinoma was defined as a contiguous sheet of malignant cells with recognizable intercellular lumina.^23^ IDC was not included in the tumour grade, and no size threshold was used for the invasive cribriform pattern. Absolute length of GP3‐5 was calculated in millimetres per core by multiplying respective GP percentages with biopsy core tumour length.

### Statistical Analysis

For statistical analyses, GG, GP4/GP5 percentage and absolute length of the biopsy core with the highest GG were used. Due to the negligible diagnostic yield of additional 7th or 8th TRUS‐targeted biopsies, we did not differentiate between systematic and TRUS‐targeted biopsies.^24^, ^25^ The relationship between absolute GP4 length and percentage was depicted by scatterplots and quantified using the Spearman correlation coefficient. We randomly assigned cT1–T4 values to four patients with missing cT‐stage. Multivariable Cox regression analyses were used to estimate prognostic impact on time to metastasis. The proportional hazards assumption was fulfilled for all models. Follow‐up was truncated after 20 years to ensure informative estimates. The baseline model included cT1 vs ≥cT2, PSA at diagnosis and percentage positive biopsies. These predictors were chosen based on clinical expertise. In a sub‐analysis, treatment (RP and radiotherapy) was included as a covariate to evaluate its effect. We did not include treatment as a covariate in the primary analysis because a previous ERSPC study had shown that differences in treatment had very little effect on PCa‐specific mortality and the number of patients included in the analysis would be lower.^26^ Non‐linear modelling of continuous predictors was assessed by fractional polynomials.^27^ The baseline model was extended by adding pathological parameters. First, we added total GP3 tumour length in all cores, and GP4 and GP5 in the highest biopsy core to investigate whether GP3 volume has independent predictive value for metastasis. We chose to include the total sum of GP3 in all biopsies to avoid multicollinearity with GP4/GP5 quantity in the biopsy with the highest GS/GG. To account for the high proportions of patients without GP4 or GP5, we included the covariates ‘GP4 Absence’ and ‘GP5 Absence’.^28^ In a new model, we also extended our baseline model with GG, highest core GP4 and GP5 tumour percentages and CR/IDC. In a sub‐analysis, treatment was included as a covariate. Discriminative ability was estimated by c‐indices. Models were internally validated using bootstrapping to correct for optimism. All statistical analyses were performed in R version 4.3.3 by a statistician (SR).

## Results

### Patient Characteristics

The cohort consisted of 1,881 men with a median age of 67 years [interquartile range (IQR) 64–71]. The median PSA level was 4.6 ng/mL (IQR 3.4–7.1) and 45% had ≥cT2 (Table S1). The median percentage of positive cores was 33% (IQR 17–50). At 20‐year follow‐up, 154 patients experienced metastasis, and 496 were still at‐risk (alive without metastasis). The probability of MFS at 20 years was 0.88 (95% CI 0.87–0.90).

### Correlation GP4 Percentage and Length

**Figure 2:**
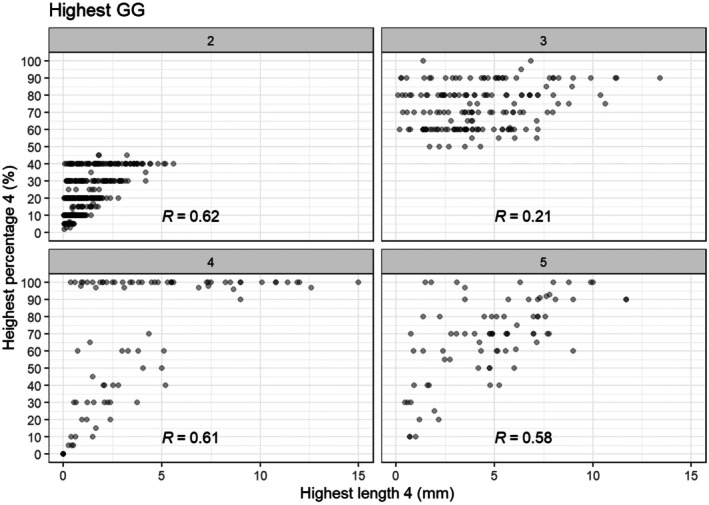
Scatterplot of the relation between percentage GP4 in highest core and absolute length GP4 in highest core in GG2 (**A**), GG3 (**B**), GG4 (**C**) and GG5 (**D**). Spearman's correlation coefficient (*R*).

Figure 2 shows the correlation between highest biopsy GP4 absolute length and percentage. In GG2 (*R* = 0.62), GG4 (*R* = 0.61) and GG5 (*R* = 0.58), the correlation between GP4 length and percentage was moderate–strong, whereas in GG3, it was weak (*R* = 0.21). The highest GP4 length did not exceed 5.6 mm in GG2, whereas in GG3, GG4 and GG5, the lengths widely ranged: 0.1–13 mm, 0–15 mm and 0.5–12 mm, respectively. The variability of GP4 length and percentage in GG4 and GG5 can be explained by the heterogeneous composition of those GGs encompassing GS 3 + 5 = 8, 5 + 3 = 8 and 4 + 4 = 8 for GG4 and GS 4 + 5 = 9, 5 + 4 = 9 and 5 + 5 = 10 for GG5.

### Predictive Value of GP3

Model coefficients of the baseline variables cT‐stage, PSA and percentage positive cores, complemented by absolute GP3, GP4 and GP5 lengths are presented in Table 1. While ≥cT2, PSA, percentage positive cores, lengths of GP4 and GP5 were all statistically significantly associated with MFS, total GP3 length was not (Hazard Ratio [HR] 0.98, 95% CI 0.96–1.01, *P* = 0.2).

**Table 1:** Hazards ratios of baseline model with absolute lengths G3–G5 in total cohort, predicting metastatic‐free survival truncated at 20 years.

Predictor	Total cohort (*N* = 1881)	*P*‐value
	HR (95% CI)	
≥cT2 vs cT1 (ref.)	1.51 (1.00–2.27)	0.05
PSA (per doubling, in ng/mL)	1.45 (1.25–1.70)	<0.001
Percentage positive cores	2.62 (1.04–6.64)	0.04
Absolute length GP3 (cm)	0.98 (0.96–1.01)	0.2
Absence length GP4	0.39 (0.24–0.63)	<0.001
Absolute length GP4 (cm)	1.18 (1.11–1.26)	<0.001
Absence length GP5	0.96 (0.57–1.63)	0.9
Absolute length GP5 (cm)	1.20 (1.08–1.33)	<0.001

CI, confidence interval; cT, clinical tumor stage; GP, Gleason pattern; HR, hazard ratio; PSA, prostate‐specific antigen.

### Comparison of Models and Addition of CR/IDC

The baseline model (Model 1) had an optimism corrected *c*‐index (OCC) of 0.790 (Table 2). Adding GG (Model 2; OCC = 0.830), absolute GP lengths (Model 3; OCC = 0.834) or GP percentages (Model 4; OCC = 0.835) to the baseline model, all resulted in better discrimination. The addition of CR/IDC improved the performance of all models; the baseline model with CR/IDC had comparable discriminative performance (Model 5; OCC = 0.847) to the baseline models with GG (Model 6, OCC = 0.847), GP lengths (Model 7, OCC = 0.848) and percentages (Model 8, OCC = 0.848) together with CR/IDC.

**Table 2:** Discriminative performance of clinicopathological models for metastasis‐free survival.

Model	Predictors	Optimism corrected *c*‐index
1. Baseline	cT, PSA, percentage positive cores	0.790
2. GG	Baseline model + GG	0.830
3. Length	Baseline model + total GP3 + highest core GP4 + highest core GP5	0.834
4. Percentage	Baseline model + highest core percentage GP4 + highest core percentage GP5	0.835
5. CR/IDC	Baseline model + CR/IDC	0.847
6. GG + CR/IDC	Baseline model + GG + CR/IDC	0.847
7. Length + CR/IDC	Baseline model + total GP3 + highest core GP4 + highest core GP5 + CR/IDC	0.848
8. Percentage + CR/IDC	Baseline model + highest core percentage GP4 + highest core percentage GP5 + CR/IDC	0.848

CR/IDC, invasive cribriform/intraductal carcinoma; cT, clinical tumour stage; GP, Gleason pattern; PSA, prostate‐specific antigen.

In the baseline model complemented with absolute GP3–GP5 lengths and CR/IDC (Model 7), total GP3 length was not a statistically significant predictor for 20‐year MFS (HR 0.99, 95% CI 0.97–1.00, *P* = 0.3), whereas PSA, GP4 length, GP5 length and CR/IDC were (Table S2). The GG categories as well as the GP4 and GP5 percentages were statistically significant predictors, both in the models with (models 6 and 8) and without CR/IDC (models 2 and 4). Table S3 presents the results of a sub‐analysis including treatment as a covariate. While treatment was a statistically significant predictor of 20‐year MFS (radiotherapy vs RP HR 2.28, 95% CI 1.40–3.70, *P* < 0.001), total GP3 length was not (HR 0.98, 95% CI 0.95–1.01, *P* = 0.2).

## Discussion

Men with GS6/GG1 PCa at RP have very low, if any, risk of developing metastasis and dying of disease, whereas those with GG1 at biopsy have low risk of aggressive disease, probably due to under‐sampling of ≥GG2.^2^, ^3^, ^4^, ^29^ While pure GP3 does not seem to have metastatic potential, it is not clear whether GP3 volume in the presence of GP4 or GP5 has additional prognostic impact.

In this biopsy study, we found that GP3 quantity in the presence of GP4 or GP5 does not have significant value in the prediction of 20‐year MFS. Although GP3 relative volume has a major impact on assigning the GS/GG, MFS seems to be related to absolute GP4 and GP5 quantity rather than their proportion to GP3 disease, which is in line with previous studies. For example, Vickers *et al*. demonstrated that adding information on GP3 volume did not meaningfully aid the prediction of adverse pathology in GG2 patients, once the total length of GP4 in biopsies was known.^9^ In a cohort of GG2–GG3 patients who underwent MRI‐targeted biopsies, Kamecki *et al*. found that absolute GP4 length was predictive for adverse pathology at RP, whereas GP4 percentage lacked significant difference.^16^

GS assignment is largely based on the relative proportion of different Gleason growth patterns; for instance, GS 3 + 4 = 7/GG2 having <50% GP4 and GS 4 + 3 = 7/GG3 having ≥50% GP4. Overall, we found a moderate correlation between the percentage and absolute length of GP4, meaning that men with a higher GP4 percentage also have a larger GP4 volume. However, scenarios exist where, for instance, low volume GG3 has less absolute GP4 than large volume GG2 biopsies. The current study does not provide an answer as to what the biological behaviour and optimal clinical management of such cases are, necessitating multidisciplinary risk stratification and further studies. Our study does show that models including GP4 and GP5 absolute length (Model 3) and percentage (Model 4) have numerically slightly better discriminative performance than a baseline model with GG only (model 2) though this difference may not be clinically significant for prognostic stratification. Several groups have studied GP4 quantification methods on biopsies, with varying results. Dean *et al*. found in 457 GG2 patients that the total length of GP4 on biopsy outperformed percentage GP4 in predicting adverse pathology.^13^ GP4 length showed an area under the curve increase of 0.044 beyond the baseline model; a number very similar to our study. Nonetheless, our study did not show a superior discriminative ability of GP4 and GP5 lengths (Models 3 and 7) compared to percentages GP4 and GP5 (Models 4 and 8) for MFS in the primary analysis. In our sub‐analysis including treatment as a covariate, we found a slightly increased discriminative ability of 0.003 of which the clinical impact is limited. Delahunt *et al*. studied 10‐year distant progression, PCa‐specific mortality and all‐cause mortality in GS7 patients.^14^ Similar to our study, they found that GP4 length and percentage had higher c‐indices than binary GG2/3 for all outcomes. They also found that the percentage of biopsied tissue that was GP4, outperformed the absolute length of GP4. However, as in our study, the c‐indices were close: the *c*‐index difference for distant progression between these predictors was 0.014. Perera *et al*. studied absolute lengths and percentages of GP4 in the highest biopsy and across all biopsies to assess postoperative biochemical recurrence at 3 years in GG2 patients.^12^ They were unable to assess differences in discriminative ability, but all methods were associated with biochemical recurrence. Since the current and other studies have not consistently shown better performance of predictive models including GP4 absolute length instead of percentage, we do not yet recommend routinely including absolute length estimates in pathology reports. However, we would recommend multidisciplinary team discussion on optimal risk stratification, especially in men with low‐volume GP4 in GG3‐4 disease.

CR/IDC status is a well‐known important factor for clinical outcome and should be reported in all PCa biopsy reports.^1^, ^30^ In our study, model performance significantly increased when CR/IDC status was considered (Model 5), after which detailed GP quantification did not further add to optimization (Models 6–8). We believe that this is not the result of overfitting but is due to MFS being the outcome of interest. Previous studies have shown that CR/IDC status has particularly strong predictive value for the development of metastasis.^17^ Other endpoints such as RP adverse pathology and biochemical recurrence seem to be significantly influenced by other pathological variables besides CR/IDC, for example, GP4 percentage.^31^ Therefore, we recommend still including GP4 quantification in pathology reports besides CR/IDC in all GS7 biopsies.^1^, ^30^

This study's strengths are the detailed revision of all biopsy specimens and using 20‐year MFS as a clinically relevant endpoint. Inherent to such long‐term outcome measures is the inclusion of men who had undergone systematic sextant biopsies at that time and did not have diagnostic magnetic resonance imaging (MRI) or targeted biopsies as is current practice, which limits the applicability of the findings to modern clinical settings. The use of MRI and targeted biopsies predominantly results in lower detection rates of GG1 PCa, also in a screening setting.^32^ On the other hand, the high variability of tissue core numbers taken in current practice, including multiple systematic, perilesional and targeted biopsies, hampers meaningful consideration of the pathological percentage of positive biopsies, while the percentage of positive cores was an interpretable and significant parameter in our study. Despite the higher risk of under‐sampling GP4 and GP5 in sextant compared to targeted biopsies, we observed that absolute GP3 length was not predictive. Furthermore, we included the absolute GP4 and GP5 lengths in the single core with the highest GG and not their total volume in all biopsies to facilitate translation to current practice with large variability in biopsy number and inclusion of targeted biopsies. Another limitation is that treatment approaches have changed since the 1990s. Most low‐risk patients in this study received active treatment, whereas active surveillance is now widely considered acceptable. Novel systemic agents and radiation modalities have emerged since, which possibly influence the generalizability of the results.

## Conclusion

GP3 volume does not have a significant impact on the prediction of 20‐year MFS in PCa biopsies once the volume of GP4 and/or GP5 is known. Although GP3 relative volume plays a significant role in the GS/GG system, MFS seems to be related to the absolute GP4 and GP5 quantity rather than their proportion towards GP3 disease. Further studies on the impact of GP4 and GP5 length in the presence or absence of CR/IDC based on contemporary biopsy practice using MRI are necessary to determine if and how absolute high‐grade tumour quantification should routinely be reported and considered in clinical decision‐making.

## Author contributions

LJK performed the research. LJK, SR, IIV and GJLHL designed the study. LJK and SR analysed the data. LJK wrote the paper. CFK, LLR, RCNB and MJR critically reviewed the manuscript. GJLHL supervised.

## Conflict of interest statement

LJK reports that the Jaap Schouten Foundation provided a grant to financially support the PhD work of LJK. The Jaap Schouten Foundation was not involved in the study contents of any kind and also does not have a conflict of interest regarding study contents or results. RCNB reports the following: advisory board (Astellas, Janssen), speaker (Amgen, Astellas, Ipsen, Janssen, MSD), travel grants (Astellas, Bayer), research support (Astellas, Janssen). The ERSPC was supported by the Dutch Cancer Society (KWF 94‐869, 98‐1657, 2002‐277, 2006‐3518, 2010‐4800), the Netherlands Organization for Health Research and Development (ZonMW‐002822820, 22000106, 50‐50110‐98‐311, 62300035), the Dutch Cancer Research Foundation (SWOP) and an unconditional grant from Beckman‐Coulter‐Hybritech Inc. The other authors report no conflicts of interest.

## Supporting information


Data S1.

## Data Availability

The data that support the findings of this study are available if requested specifically by other researchers with the intention of performing their own original research, of whom we have seen and agreed upon a data analysis plan. We would like to reserve the right to refrain from sharing data.
