# Supplementary material for: Rethinking Gleason pattern quantification in predicting metastasis: results of 20 years of follow‐up in the Rotterdam section of the European Randomized Study of Screening for Prostate Cancer
Source: Histopathology. 2025 Nov 26;88(4):881–8. doi: 10.1111/his.70052 (PMC12891930; doi:10.1111/his.70052)
Supplement: Supplementary file 1 — Data S1. [file HIS-88-881-s001.docx]

***Supplementary table 1*** *Patient characteristics of total cohort stratified by ISUP Grade Groups on highest biopsy.*

|  | Total cohort,  N = 1,881 | GG1,  N = 1,115 | GG2,  N = 431 | GG3,  N = 170 | GG4,  N = 86 | GG5,  N = 79 |
| --- | --- | --- | --- | --- | --- | --- |
| cT stage |  |  |  |  |  |  |
| T1 | 1,036 (55%) | 746 (67%) | 203 (47%) | 44 (26%) | 25 (29%) | 18 (23%) |
| T2 | 605 (32%) | 319 (29%) | 160 (38%) | 67 (39%) | 35 (41%) | 24 (30%) |
| T3 | 229 (12%) | 46 (4.1%) | 67 (16%) | 56 (33%) | 24 (28%) | 36 (46%) |
| T4 | 7 (0.4%) | 0 (0%) | 1 (0.2%) | 3 (1.8%) | 2 (2.3%) | 1 (1.3%) |
| Unknown | 4 | 4 | 0 | 0 | 0 | 0 |
| Age at diagnosis | 67 (64, 71) | 67 (63, 71) | 67 (64, 71) | 69 (65, 72) | 68 (65, 72) | 67 (64, 71) |
| PSA | 4.6 (3.4, 7.1) | 4.2 (3.2, 5.9) | 5.1 (3.6, 7.7) | 6.0 (4.4, 11) | 9.2 (4.9, 15) | 7.7 (5.3, 13) |
| Percentage positive cores | 33 (17, 50) | 25 (17, 33) | 43 (29, 57) | 50 (33, 71) | 50 (33, 71) | 57 (43, 75) |
| Total length GP3 (mm) | 3.7 (1.3, 9.3) | 2.4 (1.0, 5.6) | 8.3 (3.5, 14) | 6.5 (2.5, 14) | 5.7 (2.0, 12) | 4.8 (0.9, 11) |
| Highest core length GP4 (mm) | 0.0 (0.0, 1.0) | 0.0 (0.0, 0.0) | 0.7 (0.4-1.6) | 3.8 (2.3, 5.7) | 2.7 (1.2, 5.5) | 4.9 (2.4, 7.0) |
| Highest core length GP5 (mm) | 0.0 (0.0, 0.0) | 0.0 (0.0, 0.0) | 0.0 (0.0, 0.0) | 0.0 (0.0, 0.0) | 0.0 (0.0, 0.7) | 1.7 (0.8, 5.4) |
| Highest core percentage 4 (%) | 0.0 (0.0, 30) | 0.0 (0.0, 0,0) | 20 (10, 30) | 70 (60, 80) | 97 (30, 100) | 70 (55, 90) |
| Highest core percentage 5 (%) | 0.0 (0.0, 0.0) | 0.0 (0.0, 0.0) | 0.0 (0.0, 0.0) | 0.0 (0.0, 0.0) | 0.0 (0.0, 0.7) | 1.7 (0.8, 5.4) |
| Presence of CR/IDC | 277 (15%) | 12 (1.1%) | 72 (17%) | 100 (59%) | 45 (52%) | 47 (59%) |
| Data is displayed as numbers (%) or median (interquartile range)  Abbreviations: cT-stage (clinical tumor stage), PSA (prostate-specific antigen), GP (Gleason pattern), CR/IDC (invasive cribriform/intraductal carcinoma). | | | | | | |

***Supplementary table 2*** *Hazards ratios of baseline model with absolute lengths G3-5 in total cohort and invasive cribriform/intraductal carcinoma, predicting metastatic-free survival truncated at 20 years. Predictors included: clinical tumor stage (cT), prostate-specific antigen (PSA), percentage positive cores at sextant biopsy, total length of GP3 in all cores, GP4 and GP5 in highest biopsy, and invasive cribriform/intraductal carcinoma (CR/IDC).*

|  | Total cohort, N=1,881 |  |
| --- | --- | --- |
| Predictor | **HR (95% CI)** | **p-value** |
| ≥cT2 versus cT1 (ref.) | 1.41 (0.94-2.12) | 0.1 |
| PSA (per doubling, ng/mL) | 1.44 (1.24-1.68) | <0.001 |
| Percentage positive cores | 2.10 (0.83-5.32) | 0.12 |
| Absolute length GP3 (cm) | 0.99 (0.96-1.01) | 0.3 |
| Absence length GP4 | 0.52 (0.31-0.89) | 0.016 |
| Absolute length GP4 (cm) | 1.12 (1.05-1.20) | <0.001 |
| Absence length GP5 | 0.93 (0.55-1.57) | 0.8 |
| Absolute length GP5 (cm) | 1.18 (1.07-1.21) | 0.001 |
| CR/IDC present versus not-present (ref.) | 2.91 (1.92-4.40) | <0.001 |
| Abbreviations: cT (clinical tumor stage), PSA (prostate-specific antigen), GP (Gleason pattern), CR/IDC (invasive cribriform/intraductal carcinoma), HR (hazard ratio), CI (confidence interval). | | |

***Supplementary table 3:***

*Table 3a: Discriminative performance of clinicopathological models for metastasis-free survival, including treatment as predictor. Treatment is included as radical prostatectomy (n=641) or radiotherapy (n=505). The number of patients in this analysis is 1,146 and the number of events (metastasis) is 86.*

| Model | Predictors | c-index |
| --- | --- | --- |
| 1 Baseline | cT, PSA, percentage positive cores, treatment | 0.801 |
| 2 GG | Baseline model + GG | 0.839 |
| 3 Length | Baseline model + total GP3 + highest core GP4 + highest core GP5 | 0.851 |
| 4 Percentage | Baseline model + highest core percentage GP4 + highest core percentage GP5 | 0.848 |
| 5 CR/IDC | Baseline model + CR/IDC | 0.852 |
| 6 GG + CR/IDC | Baseline model + GG + CR/IDC | 0.855 |
| 7 Length + CR/IDC | Baseline model + total GP3 + highest core GP4 + highest core GP5 + CR/IDC | 0.860 |
| 8 Percentage + CR/IDC | Baseline model + highest core percentage GP4 + highest core percentage GP5 + CR/IDC | 0.857 |
| Abbreviations: cT (clinical tumor stage), PSA (prostate-specific antigen), GP (Gleason pattern), CR/IDC (invasive cribriform/intraductal carcinoma) | | |

*Table 3b: Hazards ratios of baseline model including treatment with absolute lengths G3-5 in total cohort, predicting metastatic-free survival truncated at 20 years. Predictors included: clinical tumor stage (cT), prostate-specific antigen (PSA), percentage positive cores at sextant biopsy, treatment (radical prostatectomy or radiotherapy), total length of GP3 in all cores, GP4 and GP5 in highest biopsy.*

|  | Total cohort, N=1,146 |  |
| --- | --- | --- |
| Predictor | **HR (95% CI)** | **p-value** |
| ≥cT2 vs cT1 (ref.) | 1.34 (0.75-1.09) | 0.3 |
| PSA (per doubling, in ng/mL) | 1.34 (1.09-1.64) | 0.005 |
| Percentage positive cores | 1.80 (0.52-6.29) | 0.4 |
| Treatment RT vs RP (ref.) | 2.28 (1.40-3.70) | <0.001 |
| Absolute length GP3 (cm) | 0.98 (0.95-1.01) | 0.2 |
| Absence length GP4 | 0.34 (0.17-0.69) | 0.003 |
| Absolute length GP4 (cm) | 1.21 (1.12-1.31) | <0.001 |
| Absence length GP5 | 1.02 (0.52-2.01) | 1.0 |
| Absolute length GP5 (cm) | 1.02 (1.00-1.03) | 0.009 |
| Abbreviations: cT (clinical tumor stage), PSA (prostate-specific antigen), RT (radiotherapy), RP (radical prostatectomy), GP (Gleason pattern), HR (hazard ratio), CI (confidence interval). | | |
